# Effect of Pesticide Exposure over DNA Damage in Farmers from Los Reyes, Michoacan in Mexico

**DOI:** 10.3390/toxics11020122

**Published:** 2023-01-26

**Authors:** Rafael Valencia-Quintana, Mirta Milić, Stefano Bonassi, Maria Antonieta Ochoa-Ocaña, Victoria Campos-Peña, Maria Guadalupe Tenorio-Arvide, Guillermo Alejandro Pérez-Flores, Juana Sánchez-Alarcón

**Affiliations:** 1 Laboratorio “Rafael Villalobos-Pietrini” de Toxicología Genómica y Química Ambiental, Facultad de Agrobiología, Universidad Autónoma de Tlaxcala, CA Genética y Ambiente UATLX-CA 223, Red Temática de Toxicología de Plaguicidas, Tlaxcala 90120, Mexico; 2 Mutagenesis Unit, Institute for Medical Research and Occupational Health, Ksaverska Cesta 2, 10000 Zagreb, Croatia; 3 Department of Human Sciences and Quality of Life Promotion, San Rafaele University, 00166 Rome, Italy; 4 Unit of Clinical and Molecular Epidemiology, IRCCS San Rafaele Pisana, 00166 Rome, Italy; 5 Unidad Académica de Estudios Regionales, Coordinación de Humanidades, UNAM, Jiquilpan 59510, Mexico; 6 Experimental Laboratory of Neurodegenerative Diseases, National Institute of Neurology and Neurosurgery Manuel Velasco Suárez, Mexico City 14269, Mexico; 7 Centro de Investigación en Ciencias Agrícolas, Benemérita Universidad Autónoma de Puebla, Puebla 72570, Mexico

**Keywords:** alkaline comet assay, DNA damage, protective equipment

## Abstract

In the municipality of Los Reyes, Michoacán, in Mexico, several economic activities coexist; however, the most relevant is agriculture. It stands out as an agro-industrial center and commercial enclave in the region, suitable for the cultivation of sugar cane; however, currently fruit growing takes first place with blackberry, raspberry and blueberry, followed by avocado, peach, strawberry and other crops. A large quantity and variety of pesticides are applied to crops, consequently the population is at constant risk. This study aimed to evaluate whether pesticides are a factor in genetic damage to agricultural workers from Los Reyes, Michoacán, using alkaline comet assay. Fifty-nine residents participated (41 workers and 18 controls). Results included confounding factors (alcohol consumption, smoking habit, gender, age, BMI, etc.) indicated a non-significant statistical difference between two groups, with higher DNA damage values in workers that was higher than the values expected in a normal healthy unexposed population. It seems that the control measures, safe handling of pesticides and quality standards, required by the producers so that their products can be exported, have resulted in less damage, despite workers’ activity, but higher damage than the reference values still requires regular surveillance of those exposed. The use of protective equipment or measures can reduce the risk of damage, so it is also necessary to promote their service and comply with labor regulations for agricultural workers.

## 1. Introduction

In the municipality of Los Reyes, Michoacan, in Mexico, several economic activities coexist; however, the most relevant is agriculture. It stands out as an agro-industrial center and commercial enclave in the region, with suitable land for the cultivation of sugar cane. However, fruit growing comes first with blackberry, raspberry and blueberry, followed by avocado, peach, strawberry and other crops. In their attempt to increase the productivity and yield of their crops, farmers have resorted to the use of pesticides [1], with the purpose of controlling pests and diseases. To this day, they are the most effective form of protecting these crops [2]. Nevertheless, given pesticides’ toxic properties [3], despite their benefits, these substances represent a risk to both the environment and to humans.

As in most developing countries, farmers in Mexico need more knowledge of the risk to health derived from the mishandling of pesticides during their preparation and spraying in crop fields. Both poor handling and application practices of pesticides cause occupational and/or environmental exposure to these agents [4,5] that can enter exposed individuals through different routes (inhalation, topical and ingestion). They can affect the functioning of various organs and/or systems (nervous, endocrine, reproductive and immune, among others) [6]. The use of pesticides has also been associated with genetic damage [2,7,8,9]. Though most biomonitoring and genotoxicity research have been done in advanced nations, they are most relevant to emerging nations. In Mexico, around 7 million people work in agriculture and it is estimated that 25.4% of its population may be directly exposed to pesticides. Despite this, few studies have been developed in Mexico and in some cases their results have been contradictory [8]. Thus, the purpose of the present study is to estimate the possible effects caused by exposure to complex mixtures of pesticides in agricultural workers despite strict standards of protection and hygiene in the handling of pesticides in the Municipality of Los Reyes, State of Michoacán, in Mexico, through the analysis of genetic damage using the comet assay or single-cell alkaline electrophoresis assay in whole blood.

## 2. Materials and Methods

All chemicals and materials were from Sigma Aldrich, St. Louis, MI, USA, unless another source is mentioned.

### 2.1. Sample Collection and Study Population

This research was approved by the State Research Ethics Committee of the Tlaxcala State Bioethics Commission (CI-CEI-01/2018). The participants signed an informed consent form and responded to a structured inquiry form to gather information on socio-demographic features, confounding discriminatory factors, details of pesticide management, use of personal protective measures (PPM) and medication.

Fifty-nine individuals from Los Reyes municipality in the state of Michoacan, Mexico, with an age range of 18–72 years, were considered in this research and were divided into two groups, i.e., 41 agricultural workers and 18 individuals as a control group from the same geographic area. Control group was without exposure to any genotoxic agent, nor labor activity related to agriculture. The lower number of subjects in the control group is due to a higher proportion of subjects who decided not to sign the informed consent. Furthermore, some subjects after the entry interview no longer satisfied the inclusion criteria (mainly due to agricultural work). The lower proportion of men in the control group is mostly due to the difficulty in finding subjects unexposed and not working in agriculture, a condition most likely for women.

One sample of total blood was collected from each farmer or control subject into vacutainers tube, with EDTA as an anticoagulant (4 mL).

### 2.2. Comet Assay

In the procedure described by Speit and Rothfuss (2012) [10], comet assay in alkaline conditions was performed with whole blood, with EDTA as an anticoagulant. Minimum Information for Reporting Comet Assay rules (MIRCA) were applied with some modifications [11]. Zeiss microscope connected with Comet Assay IV image analysis system (Instem, London, UK) was used. Additional details of methodology can be found in a previous study [9].

### 2.3. Statistical Analysis

Descriptive statistics (mean, standard deviation, standard error), independent samples Student’s t and χ^2^ to evaluate the data of sociodemographic features, and Yate’s correction tests were used. For comet assay parameters, statistical analysis was performed by Mann Whitney U test.

## 3. Results

### 3.1. Sociodemographic Features of the Study Groups

The control group comprised 18 subjects between 21 and 85 years (43.56 ± 4.02 (mean age ± standard deviation (SD)). The farmers group included 41 exposed subjects aged between 23 and 73 years (40.98 ± 2.24). Demographic features of the populations studied (control and farmers) are shown in Table 1. There were no differences in age distribution between the control and the exposed group (t = 0.60, df = 57.0, *p* = 0.55), nor for BMI and gender (female t = 0.42, df = 18, *p* = 0. 68; male t = 1.37, df = 17, *p* = 0.17). Comparing proportional frequencies of smokers and non-smokers between groups, there were statistically significant differences (χ^2^ = 13.42, df = 1, *p* = 0.0002), with more smokers in the control group, and interestingly the same data was reported for alcohol intake. Most volunteers described the use of personal protective measures (90%).

Farmers were regularly in contact with insecticides (81%), fungicides (11%) and herbicides (8%); in particular exposure to organophosphates (35%), carbamates (15%), pyrethroids (15%), organochlorines (12%) and others (23%) was reported. The activities reported by the exposed group included spraying, cultivating and harvesting, which were carried out by all of them, often causing exposure to mixtures of pesticides (Table 2). According to the WHO (2020), USEPA (2018) and IARC (2020) [12,13,14], these compounds are categorized from slightly to highly hazardous. Four of these have been classified in Group 2A (probably carcinogenic to humans) (aldrin metabolite, diazinon, glyphosate and malathion), two classified in Group 2B (possibly carcinogenic to humans) (mancozeb and parathion) and one classified in Group 3 (may not be carcinogenic to humans) (dicofol) by IARC. On the other hand, the WHO categorizes these compounds as extremely hazardous (oxamyl and parathion), highly dangerous (abamectin, azinphos-ethyl, carbofuran, cyfluthrin, methamidophos and methomyl), moderately hazardous (acephate, chlorpyrifos, diazinon, dicofol, dimethoate, endosulfan, imidacloprid, lambda-cyhalothrin, paraquat, permethrin, pyrethrin and zeta-cypermethrin) and slightly hazardous (diuron, glyphosate, malathion and thiabendazole).

### 3.2. Comet Assay

Table 3 shows the results of primary DNA damage. In the exposed group, the values of tail length, intensity, moment and Olive moment were 22.89 ± 1.29 µm, 12.55 ± 1.23%, 1.44 ± 0.25 and 1.02 ± 0.34, respectively. Values in control group were 20.58 ± 1.04 µm, 9.51 ± 0.83%, 0.90 ± 0.10 and 0.80 ± 0.09, respectively. Values in the exposed group showed slight increases; however, the differences found were not statistically significant (*p* > 0.05). In the same way, when the exposed group was divided based on duration of work (<6 and > 6 years), use of protective equipment (PPM), age (<35 and >35 years), gender and smoking and drinking habits, no significant differences were found (*p* > 0.05) in the comet parameters between different groups of variables tested. However, it is valuable to mention that our group previously established reference comet assay values for healthy, cancer free, cancer prone and deceased (in future) values that are mostly used only for tail intensity parameter and, according to those values, the exposed group still has higher values than expected in a normal healthy population, pointing out that these exposed groups still need regular surveillance [15,16].

## 4. Discussion

To increase productivity and avoid losses due to pests, farmers resort to the application of pesticides. For this reason, pesticides have become a factor in food security. However, their use can affect non-target organisms, including humans, due to their indiscriminate and uncontrolled use [17]. Many health problems have been correlated to pesticide contact [18] and DNA damage. In some countries, like Mexico, there is a lack of information on actual human exposure to different pesticides [19] and effects at the genetic level in populations occupationally exposed to these compounds [8].

Pesticide exposure represents a high risk. An integral approach for exposure assessment is human biomonitoring (HBM) [20]. Genotoxic assessment to determine health risks from occupational contact to complex mixtures of these compounds in humans has proven to be a valuable and achievable means [21,22].

Different pesticides, including organochlorines, organophosphorus, carbamates and pyrethroids, are the most commonly used in developing countries [23]. These groups represent 77% of the compounds used in Los Reyes, Michoacán, Mexico.

The comet assay can assess occupational exposure to pesticides [24]. This bioassay was conducted in the present work to evaluate DNA damage in agricultural workers from Los Reyes Michoacán in Mexico. The parameters considered were the comet tail length, the intensity, the moment and the Olive moment, which are the most frequently used [21], Tail intensity is generally accepted as the most reliable endpoint to measure DNA damage with the comet assay. Nevertheless, in explorative studies like the present one, alternative endpoints may report different results and open alterative headways to explore, searching for specific mechanisms of specific exposures [15,16].

The results showed a slightly higher level of DNA damage than in those who do not perform these tasks. However, this difference was not statistically significant.

As in the present study, Pastor et al. (2001) [25] found no differences in the frequencies of cytogenetic damage between exposed and control individuals in both buccal cells and peripheral blood lymphocytes, in farmers exposed to pesticides, using the micronuclei test.

Contrary to this, other studies have reported an increment in DNA injury induced by exposure to pesticides [2,21,22,26,27,28]. In these cases, a low awareness of pesticide risk has been assumed [29].

Still, we need to mention that, according to our newest results, although the workers’ groups did not have significantly higher DNA damage values, they still had higher values than those established for the healthy population, even reaching the values of the group with genomic damages but cancer free (tail intensity up to 9% for normal population, up to 12.4% for cancer free population, around 17% for cancer prone and above 18% for deceased population from mortality registry) [15,16]. This points to the fact that workers should have, in the future, further regular surveillance, and awareness of using protective equipment in most (90%) should be encouraged as results demonstrate in this study (in the shape of slightly higher DNA damage values).

The single cell gel electrophoresis assay has been applied in environmental and occupational human assessment, in studies on genotoxicity due to exposure to potentially mutagenic agents, at the clinical level, because of lifestyle, or due to the interaction between diet and antioxidant consumption on carcinogenesis, analysis of irradiated foods in ecotoxicology, radiation biology, environmental genotoxicity and genetic toxicology [30].

The specific manifestation of pesticides’ effects and their intensity depend on the type of pesticide, its concentration, the exposure time and individual susceptibility to other factors [3,31]. It is noteworthy that not all people exposed to genotoxic agents respond similarly. While some people can present carcinogenesis processes, others are resistant to the activity of the harmful substance. This is the result of the great interindividual variability that lies in the ability to inactivate or activate potentially genotoxic and carcinogenic compounds, which is probably influenced by the polymorphisms of the genes that encode the enzymes of the xenobiotic metabolism [32] of phase I (activation) and phase II (inactivation), in addition to influencing the responsiveness of the nucleotide repair cleavage system [33]. Different studies have managed to identify genotypes associated with an increased risk of cancer due to exposure to genotoxic agents [6].

Some studies with exposed populations to pesticides find genetic damage, while others show negative results [25]. The reason for these controversial results may be that these types of studies are difficult to repeat because each exposed population presents very particular characteristics, from the different climatic conditions of the region in which they live, to lifestyle and the use of various mixtures of pesticides. In Los Reyes, Michoacán, Mexico, the farmers monitored used 26 different pesticides; the most frequently mentioned were three organophosphates (malathion (16%), parathion (15%) and paraquat (11%)) and a pyrethroid (lambda-cyhalothrin (8%)), one of these being extremely dangerous (parathion), two moderately complex (paraquat and lambda-cyalothrin) and one slightly dangerous (malathion), according to the WHO classification (WHO 2020) [12]. The level of exposure to these pesticides in Los Reyes, Michoacán, is potentially significant and data on its genotoxicity is available (malathion [34,35], parathion [36,37,38], paraquat [39,40], lambda-cyhalothrin [41,42]); however, information on genotoxic effects of complex mixtures is lacking [25].

Due to the lack protective measures during the application of pesticides farmworkers are at a high risk of damage [22,43,44,45]. Personal protective equipment (PPE) has been considered a vital safety shield against pesticide-related health risks [46]. According to the information provided by the farmers who participated in this project, most of them use some protection such as gloves, masks, goggles, or overalls (90%). The results found in this study agree with those reported by Pastor et al. (2001) [25] in workers that used more than one protective measure, where they statistically insignificant differences were reported in the frequencies of DNA damage between control and individuals exposed to complex mixtures of pesticides. Although this may explain the lack of cytogenetic damage in agricultural workers from Los Reyes, Michoacán in Mexico, it cannot be affirmed that the pesticides did not induce any adverse effect. In this particular case, no significant increase in cytogenetic damage was found when assessed by the comet assay in lymphocytes. However, as in other agricultural regions [47], an increase in the incidence of cancers of different types has been reported in Los Reyes, Michoacán, Mexico and the population has associated it with exposure to pesticides (personal information). Even in the absence of other signs of acute toxicity, cancer risk may rise significantly among exposed farmworkers [48]. The higher risk of developing cancer in agricultural workers exposed to pesticides has been supported by different epidemiological studies [22,49]. The International Agency for Research on Cancer (IARC) has classified parathion, malathion, glyphosate and diazinon as probable human carcinogens [50,51], which the participants in this study used.

In addition to the use of ecotoxicity, cytotoxicity and genotoxicity tests with the aim of assessing the potential risks from contact with pesticides at different levels of biological organization [52], the application of new molecular approaches to contribute to the elucidation of possible mechanisms through which pesticides induce different diseases, including cancer, is recommended. Deregulation of miRNAs expression (let-7, miR-9, miR-21, miR-30, miR-126 miR-155, miR-181, miR-223, miR-363 and miR-320), has been evaluated to be a leading biomarker in evaluating the response to pesticide exposure [53]. Some of these miRNAs are essential for cellular proliferation, differentiation and transformation or are targets of tumor repressors and have an essential role in cancer development [53,54]. Improving understanding of miRNA deregulation by pesticide contact will permit their proposal as biomarkers, which could improve knowledge of the molecular toxicity of pesticides.

## 5. Conclusions

It is important to promote the use of protection measures, as well as compliance with labor regulations, for risk prevention in agricultural workers, which minimize the risks of exposure to pesticides.

It is essential to conduct biomonitoring studies to determine and guarantee good working conditions for farmers, as well as providing greater awareness of the use of PPE.

In order to evaluate the association between occupational exposure to pesticides and the incidence of DNA damage, or their effects on miRNA profiles in agricultural workers, it is necessary to identify novel biomarkers of occupational pesticide exposure, so other systematic studies are needed.

## Figures and Tables

**Table 1:** Characteristics of the control and the exposed group.

Characteristics	Control Group		Exposed Group	
*n*	18		41	
Gender (M/F) (%)	6/12	(33.33/66.67)	33/8	(80.49/19.51)
Age (years, mean ± SD) (range)	43.56 ± 4.02	(21-85)	40.98 ± 2.24	(23–73)
BMI (kg/m^2^, mean ± SD) (range)	25.73 ± 0.81	(20.79-32.44)	29.09 ± 0.94	(14.52–41.73)
Exposure time (in years, mean ± SD) (range)	NA		7.07 ± 0.51	(1 ≥ 10)
Smoking				
Smokers, *n* (%)	7	(38.89)	6	(14.63)
Non-smokers, *n* (%)	11	(61.11)	35	(85.37)
Alcohol intake				
yes, *n* (%)	7	(38.89)	6	(14.63)
no, *n* (%)	11	(61.11)	35	(85.37)
PPM, *n* (%)	NA		37 of 41	(90)

*n*-number of subjects, M-male, F-female, SD-standard deviation, BMI-body mass index, PPM-personal protective measures (equipment and procedure), NA-not applicable.

**Table 2:** List of the more frequent pesticides used by the exposed group and their hazard classification.

P	CC	Compound	IUPAC Name	WHO	USEPA/IARC
I	Carbamate	Carbofuran (Furadan)	2,2-Dimethyl-2,3-dihydro-1-benzofuran-7-yl methylcarbamate	Ib	NLC/NE
		Methomyl (Lannate)	(E,Z)-methyl N-{[(methylamino)carbonyl]oxy}ethanimidothioate	Ib	Group E/NE
		Oxamyl (Vydate)	Methyl 2-(dimethylamino)-N-[(methylcarbamoyl)oxy]-2-oxoethanimidothioate	Ia	Group E/NE
	Glycoside	Abamectin (Agrimec)	Mix of: (10E,14E,16E)-(1R,4S,5′S,6S,6′R,8R,12S,13S,20R,21R,24S)-6′-[(S)-sec-butyl]-21,24-dihydroxy-5′,11,13,22-tetramethyl-2-oxo-(3,7,19-trioxatetracyclo [15.6.1.14,8.020,24]pentacosa-10,14,16,22-tetraene)-6-spiro-2′-(5′,6′-dihydro-2′H-pyran)-12-yl 2,6-dideoxy-4-O-(2,6-dideoxy-3-O-methyl-α-L-arabino-hexopyranosyl)-3-O-methyl-α-L-arabino-hexopyranoside and (10E,14E,16E)-(1R,4S,5′S,6S,6′R,8R,12S,13S,20R,21R,24S)-21,22-dihydroxy-6′-isopropyl-5′,11,13,22-tetramethyl-2-oxo-(3,7,19-trioxatetracyclo [15.6.1.14,8.020,24] pentacosa-10,14,16,22-tetraene)-6-spiro-2′-(5′,6′-dihydro-2′H-pyran)-12-yl 2,6-dideoxy-4-O-(2,6-dideoxy-3-O-methyl-α-L-arabino-hexopyranosyl)-3-O-methyl-α-L-arabino-hexopyranoside.	Ib	NE/NE
	Imide	Imidacloprid	(EZ)-1-(6-cloro-3-piridilmetil)-N-nitroimidazolidin-2-ilidenoamina	II	Group E/NE
	Organochlorine	Aldrin	1,2,3,4,10,10-Hexachloro-1,4,4a,5,8,8a-hexahydro-1,4:5,8-dimethanonaphthalene	O	Group B2/Group 2A
		Dicofol (Kelthane)	2,2,2-trichloro-1,1-bis(4-chlorophenyl)ethanol	II	Group C/Group 3
		Endosulfan	6,7,8,9,10,10-Hexachloro-1,5,5a,6,9,9a-hexahydro- 6,9-methano-2,4,3-benzodioxathiepine-3-oxide	II	NLC/NE
	Organophosphate	Acephate (Orthene)	N-(Methoxy-methylsulfanylphosphoryl)acetamide	II	Group C /NE
		Azinphos-ethyl (Gusathion)	O,O-Diethyl S-[(4-oxo-1,2,3-benzotriazin-3(4H)-yl)methyl] phosphorodithioate	1b	NE/NE
		Chlorpyrifos (Lorsban)	O,O-Diethyl O-3,5,6-trichloropyridin-2-yl phosphorothioate	II	Group E/NE
		Diazinon	O,O-Diethyl O-[4-methyl-6-(propan-2-yl)pyrimidin-2-yl] phosphorothioate	II	NLC /Group 2A
		Dimethoate	O,O-dimethyl S-[2-(methylamino)-2-oxoethyl] dithiophosphate	II	Group C/NE
		Malathion	Diethyl 2-[(dimethoxyphosphorothioyl)sulfanyl]butanedioate	III	SEC/Group 2A
		Methamidophos (Tamaron or Tramophos)	O,S-Dimethyl phosphoramidothioate	Ib	NLC/NE
		Parathion (Folidol)	O,O-Diethyl O-(4-nitrophenyl) phosphorothioate	Ia	Group C/Group 2B
	Pyrethrin	Pyrethrin	(Z)-(S)-2-metil-4-oxo-3-(penta-2,4-dienil)ciclopent-2-enil (1R,3R)-2,2- dimetil-3-(2-metilprop-1-enil)ciclopropancarboxilato	II	NLC/NE
	Pyrethroids	Cyfluthrin (Baytroid)	(RS)-α-ciano-4-fluoro-3-fenoxibencil (1RS,3RS;1RS,3SR)-3-(2,2- diclorovinil)-2,2-dimetilciclopropanocarboxilato	Ib	NLC/NE
		Lambda-cyhalothrin (Karate)	(R)-α-cyano-3-phenoxybenzyl (1S)-cis-3-[(Z)-2-chloro-3,3,3-trifluoropropenyl]-2,2-dimethylcyclopropanecarboxylate and (S)-a-cyano-3-phenoxybenzyl (1R)-cis-3-[(Z)-2-chloro-3,3,3-trifluoropropenyl]-2,2-dimethylcyclopropanecarboxylate	II	Group D/NE
		Permethrin	3-fenoxibencil (1RS,3RS,1RS,3SR)-3-(2,2-diclorovinil)-2,2- dimetilciclopropancarboxilato	II	SEC/NE
		Zeta-cypermethrin (Mustang Max)	Mix of (S)-α-cyano-3-phenoxybenzyl (1RS,3RS;1RS,3SR)-3-(2,2-dichlorovinyl)-2,2-dimethylcyclopropanecarboxylate	II	NLC/NE
H	Organophosphate	Paraquat	1,1′-Dimethyl-4,4′-bipyridinium dichloride	II	Group C/NE
	Phosphonomethylglycine	Glyphosate	N-(fosfonometil)glicina-isopropilamina (1:1) o isopropilaminio N-(fosfonometil)glicinato	III	NLC/Group 2A
F	Benzimidazole	Thiabendazole (Tecto-60)	2-(tiazol-4-il) benzimidazol	III	SEC/NE
	Carbamate	Mancozeb (Manzate)	Zinc;manganese(2±);N-[2-(sulfidocarbothioylamino)ethyl]carbamodithioate	U	Group B/Group 2B
	Ureic	Diuron	3-(3,4-Diclorofenil)-1,1-dimetilurea	III	Known/Likely carcinogen/NE

I-insecticides, H-herbicides, F-fungicides, P-pesticides, CC-chemical class, NE-not evaluated, WHO classification: Ia = Extremely hazardous; Ib = Highly hazardous; II = Moderately hazardous; III = slightly hazardous; U = Unlikely to present acute hazard in normal use; FM = Fumigant, not classified; O = Obsolete as pesticide, not classified (WHO 2020). USEPA Cancer Classification: NLC, Not Likely to be Carcinogenic to Humans; SEC, Suggestive Evidence of Carcinogenicity; Group B2 Probable Human Carcinogen; Group C Possible Human Carcinogen; Group D--Not Classifiable as to Human Carcinogenicity; Group E Evidence of Non-carcinogenicity for Humans (USEPA 2018). IARC: Group 1, Carcinogenic to humans; Group 2A, Probably carcinogenic to humans; Group 2B, Possibly carcinogenic to humans; Group 3, Not classifiable as to its carcinogenicity to humans (IARC 2020) [12,13,14].

**Table 3:** Human biomonitoring of agricultural workers from Los Reyes in Michoacan Mexico, with Comet assays.

Assay/Parameters	Control Group (*n* = 18)	Exposed Group (*n* = 41)	Level of Significance (p)
Tail Length	20.58 ± 1.04	22.89 ± 1.29	0.271
Tail Intensity	9.51 ± 0.83	12.55 ± 1.24	0.262
Tail Moment	0.90 ± 0.10	1.44 ± 0.25	0.169
Olive Tail Moment	0.80 ± 0.09	1.02 ± 0.34	0.220

Values represent Mean ± SE. Statistical analysis was done by Mann Whitney U test.

## Data Availability

Not applicable.
